# An Erythroid Differentiation Signature Predicts Response to Lenalidomide in Myelodysplastic Syndrome 

**DOI:** 10.1371/journal.pmed.0050035

**Published:** 2008-02-12

**Authors:** Benjamin L Ebert, Naomi Galili, Pablo Tamayo, Jocelyn Bosco, Raymond Mak, Jennifer Pretz, Shyam Tanguturi, Christine Ladd-Acosta, Richard Stone, Todd R Golub, Azra Raza

**Affiliations:** 1 Broad Institute of Harvard and Massachusetts Institute of Technology, Cambridge, Massachusetts, United States of America; 2 Dana-Farber Cancer Institute, Harvard Medical School, Boston, Massachusetts, United States of America; 3 Brigham and Women's Hospital, Department of Medicine, Boston, Massachusetts, United States of America; 4 St. Vincent's Comprehensive Cancer Center, New York, New York, United States of America; 5 Childrens's Hospital, Boston, Massachusetts, United States of America; 6 Howard Hughes Medical Institute, Chevy Chase, Maryland, United States of America; Fred Hutchison Cancer Research Center, United States of America

## Abstract

**Background:**

Lenalidomide is an effective new agent for the treatment of patients with myelodysplastic syndrome (MDS), an acquired hematopoietic disorder characterized by ineffective blood cell production and a predisposition to the development of leukemia. Patients with an interstitial deletion of Chromosome 5q have a high rate of response to lenalidomide, but most MDS patients lack this deletion. Approximately 25% of patients without 5q deletions also benefit from lenalidomide therapy, but response in these patients cannot be predicted by any currently available diagnostic assays. The aim of this study was to develop a method to predict lenalidomide response in order to avoid unnecessary toxicity in patients unlikely to benefit from treatment.

**Methods and Findings:**

Using gene expression profiling, we identified a molecular signature that predicts lenalidomide response. The signature was defined in a set of 16 pretreatment bone marrow aspirates from MDS patients without 5q deletions, and validated in an independent set of 26 samples. The response signature consisted of a cohesive set of erythroid-specific genes with decreased expression in responders, suggesting that a defect in erythroid differentiation underlies lenalidomide response. Consistent with this observation, treatment with lenalidomide promoted erythroid differentiation of primary hematopoietic progenitor cells grown in vitro.

**Conclusions:**

These studies indicate that lenalidomide-responsive patients have a defect in erythroid differentiation, and suggest a strategy for a clinical test to predict patients most likely to respond to the drug. The experiments further suggest that the efficacy of lenalidomide, whose mechanism of action in MDS is unknown, may be due to its ability to induce erythroid differentiation.

## Abstract 2

Using gene expression profiling, Azra Raza and colleagues identified a molecular signature that predicts response to lenalidomide in patients without Chromosome 5q deletions, which suggests that these patients have a defect in erythroid differentiation.

## Editors' Summary

**Background.:**

Myelodysplastic syndrome (MDS) is a group of disorders in which the bone marrow (the spongy material found inside bones) does not make enough healthy blood cells. Normally, immature cells in the bone marrow called hematopoietic stem cells mature (differentiate) into three types of blood cells: red blood cells (which carry oxygen around the body; people with too few red blood cells are “anemic”), white blood cells (which fight off infections), and platelets (which prevent bleeding by forming blood clots). In patients with MDS, the production of these mature cell types is defective. In addition, immature cells called leukemic blasts sometimes accumulate in the bone marrow and blood. Thus, although MDS itself is not a type of cancer, it often develops into leukemia (blood cancer). The cause of most cases of MDS, which affects mainly elderly people, is not known. Its symptoms include tiredness and breathlessness (signs of anemia), frequent infections, and easy bruising or bleeding. Patients are usually given supportive care to relieve their symptoms (for example, blood transfusions to top up their red blood cells). Chemotherapy can sometimes delay the progression of MDS to leukemia and a few patients can be helped with bone marrow transplantation.

**Why Was This Study Done?:**

Recently, researchers have discovered that some people with MDS respond very well to a drug called lenalidomide. Three-quarters of patients whose MDS is characterized by the loss of a small part of Chromosome 5 need fewer blood transfusions after being given lenalidomide but only a quarter of people without this chromosomal defect respond to the drug. Unfortunately, most patients with MDS do not have this chromosome abnormality and there is no way to predict which of these patients are likely to respond to lenalidomide. Lenalidomide is a toxic drug that damages white blood cells and platelets, so it is important not to give it to people who might not benefit. In this study, the researchers have used gene expression profiling (a technique that catalogs all the genes expressed by a cell) to try to develop a way of predicting who will respond to lenalidomide

**What Did the Researchers Do and Find?:**

The researchers obtained pre-treatment bone marrow samples from patients enrolled in two clinical trials of lenalidomide and compared the gene expression profiles of the bone marrow cells from the patients who subsequently responded to the drug with the profiles of cells from nonresponding patients. In all, 47 genes were more highly expressed in nonresponders than in responders. The researchers then asked whether the expression of any gene sets (collections of genes that code for proteins that work in a single pathway) was greater in the nonresponders than in the responders. This analysis revealed a “signature” of lenalidomide response consisting of a set of genes normally expressed during the differentiation of red blood cells (an “erythroid differentiation signature”). Decreased expression of this signature was associated with a response to lenalidomide in an independent set of patients (validation set). The researchers then used the response signature and the original set of samples to develop a single score that could distinguish individual responders from nonresponders. This score accurately predicted the response of three-quarters of the patients in the validation set to lenalidomide. Finally, the researchers showed that lenalidomide promotes the erythroid maturation of normal human hematopoietic stem cells grown in dishes and stimulates the expression of the lenalidomide response signature in these cells.

**What Do These Findings Mean?:**

These findings indicate that patients with MDS who respond to lenalidomide have defective red blood cell differentiation. In addition, they suggest that it might be possible to use the response signature to develop a test that can predict which patients with MDS will benefit from treatment with lenalidomide. However, the preliminary predictive test described here will need to be tested in many more patients before it can be used as a routine clinical test. Finally, the researchers' last experiment suggests that lenalidomide may help people with MDS because it induces red blood cell differentiation. Lenalidomide therapy might, therefore be useful in other disorders in which red blood cell maturation is defective, including some forms of anemia.

**Additional Information.:**

Please access these Web sites via the online version of this summary at http://dx.doi.org/10.1371/journal.pmed.0050035.

- See a related *PLoS Medicine* Perspective article
- The US National Cancer Institute provides information for patients about myelodysplastic syndrome (in English and Spanish)
- The UK charity Cancerbackup provides information about myelodysplastic syndrome
- The American Cancer Society and the Leukemia and Lymphoma Society provide additional information about myelodysplastic syndrome
- The US Food and Drug Administration provides information for patients on lenalidomide

## Introduction

Myelodysplastic syndrome (MDS) is an acquired, neoplastic disorder of hematopoietic stem cells characterized by ineffective and dysplastic hematopoietic differentiation and a high rate of progression to acute leukemia. Therapeutic options for patients with MDS are limited: bone marrow transplantation has curative potential but is only an option for a small minority of patients, high-dose chemotherapy has poor efficacy and high toxicity, and other pharmacological therapies have, until recently, had limited benefit [1,2]. The thalidomide derivative lenalidomide (Revlimid, CC-5013), however, has recently demonstrated promise for the treatment of MDS [3,4]. Strikingly, lenalidomide response is most common in patients with deletions of the long arm of Chromosome 5. Specifically, a recent Phase II study of the activity of lenalidomide in patients with low- or intermediate-1–risk MDS and 5q deletions, showed that 76% of patients had a decreased need for blood transfusions and 61% achieved complete resolution of cytogenetic abnormalities [3]. This effect represents the most significant clinical response ever seen in MDS.

Unfortunately, the vast majority of MDS patients (over 80%) lack 5q deletions, and only 26% of such patients respond to lenalidomide [5]. It is not clear which patients without 5q deletions should receive the drug, particularly given the toxicities of lenalidomide (primarily neutropenia and thrombocytopenia), which are severe in some cases [3–5]. In order to limit exposure of lenalidomide to the patients who are most likely to benefit, a biological predictor of response is needed. Finding such predictors is particularly challenging because both the mechanism of lenalidomide activity in MDS and the molecular basis of MDS are unknown. We took a genome-wide view of the problem and used gene expression profiling to develop a predictor of lenalidomide response.

## Materials and Methods

### Patient Samples

All samples were obtained from patients treated in Phase II clinical trials, MDS-002 and MDS-003, studying the efficacy of lenalidomide in low-risk, transfusion-dependent MDS in patients without 5q deletions or with 5q deletions, respectively [3,5]. Mononuclear cells from each pretreatment bone marrow aspirate were obtained following informed consent from the patient under institutional review board–approved protocols from Rush Medical School, the University of Massachusetts Medical School, or the Dana-Farber Cancer Institute. Mononuclear cells from bone marrow aspirates were purified by Ficoll gradient. Cell pellets were dissolved in Trizol (Invitrogen) and stored at −80 °C (samples from Rush Medical School and the University of Massachusetts), or were stored in liquid nitrogen as cell pellets (samples from the Dana-Farber Cancer Institute). All available samples from these institutions were included in this study. No samples were excluded on the basis of clinical parameters. An initial training set comprised 16 samples from patients without 5q deletions. An independent test set comprised 13 samples from patients without 5q deletions. In addition, we analyzed a set of 13 samples from patients with 5q deletions.

All patients had low- or intermediate-1–risk MDS as determined by the International Prognostic Score [6], transfusion-dependent anemia requiring greater than two units of red blood cells every 8 wk, an absolute neutrophil count greater than 500/ml, and a platelet count greater than 30,000/ml. Patients with chronic myelomonocytic leukemia (CMML) with a white blood count greater than 12,000/ml were excluded. Patients in this study had not received hematopoietic growth factors for 14 d prior to the pretreatment bone marrow aspirate. Patients were treated with lenalidomide at a dose of 10 mg/d or 10 mg/d for 21 d in 4-wk cycles. Hematologic response was determined using a modified version of the criteria defined by an international working group [3,7]. A complete hematologic response was defined as transfusion independence, the absence of transfusions for 8 wk, and an increase in hemoglobin of greater than or equal to 1.0 g/dl. A minor hematologic response was defined as a 50% decrease in the number of transfusions that was sustained for eight consecutive weeks. Responders, in this study, included patients with either a minor or complete hematologic response to lenalidomide.

### Oligonucleotide Microarrays

RNA was purified from mononuclear cells using Trizol (Invitrogen). Linear amplification of 20 ng of total RNA was performed using the Ovation Biotin RNA Amplification and Labeling System (Nugen). Fragmented, labeled cDNA was hybridized to Affymetrix oligonucleotide microarrays as described previously [8,9]. Patient samples for the training set were profiled on HG_U133 Plus 2.0 microarrays; samples for the test set and samples from cells cultured in vitro were profiled using the Affymetrix HTA platform with hybridization to HG_U133AAofAv2 microarrays. Raw expression values were normalized using Robust Multiarray Averaging (RMA) [10]. Microarrays are listed in Table S1, and the complete dataset is available at http://www.broad.mit.edu/cancer/pub/Revlimid.

### Marker Gene Selection

Raw gene expression values were preprocessed and normalized using Robust Multiarray Averaging (RMA) [10]. Genes with minimal variation across the dataset were excluded by discarding genes for which the maximum gene expression value divided by the minimum value across all samples was less than two, or if the difference between maximum and minimum values was less than 100. Marker genes were ranked using the signal-to-noise metric [9]. For gene *x*, the signal-to-noise metric, *S_x_*, is calculated as:

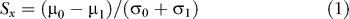
where μ_0_ and σ_0_ are the mean and standard deviation for gene *x* in class 0, and μ_1_ and σ_1_ are the respective values for class 1. Statistical significance was determined by random permutation of the class labels [9]. Significant markers were selected using a false-discovery rate (FDR) threshold of 0.1 computed using the Benjamini and Hochberg procedure [11]. Analyses were implemented using the GenePattern software package [12] using the ComparativeMarkerSelection module [13].

### Gene Set Enrichment Analysis

Gene set enrichment analysis (GSEA) was performed as described previously [14]. The dataset was converted from probe sets to gene symbols and analyzed using the java GSEA package. The input gene set database was comprised of the curated gene sets (c2) of the Molecular Signature Database version 2 (MSigDBv2) [14,15]. All gene sets, including both curated gene sets from MSigDBv2 and the response signature, are available in Table S4. Gene sets enriched in responder or nonresponder classes were ranked by normalized enrichment score, and statistical significance was determined by permutation of the gene tags.

### Gene Expression–Based Lenalidomide Response Predictor

A predictor of lenalidomide response was created by comparing the response signature to a reference signature for each sample. The response signature was defined as the marker genes of nonresponders compared to responders in the training dataset (FDR ≤ 0.10) that are present on both the U133 Plus 2.0 and U133AAofAv2 microarrays, and that increased in expression during terminal erythroid differentiation. The reference signature was defined by taking a list of previously published control genes that are invariant in other gene expression datasets [16], and selecting the five genes with the smallest standard deviation across samples in the training dataset. The five genes in the reference signature are *ACTB*, *B2M*, *GAPDH*, *TXNRD1*, and *UBE2D2*.

In order to predict response to lenalidomide using gene expression data acquired on different microarray platforms, gene expression values for each sample were replaced by their ranks among all features in that sample and then linearly scaled to the [0, 10,000] range, as described previously [17]. The expression of each gene in the erythroid signature was divided by the average gene expression for the reference signature in the corresponding sample. A *z*-score was obtained for each gene in the response signature. A single predictor score was obtained for each sample by averaging the *z*-scores for each gene in the response signature. A binary regression linear model was fit to the predictor score to determine the location of the class boundary between responders and nonresponders using the “glm” function in R [18]. To predict response in the test set of patients without 5q deletions or in the set of patients with 5q deletions, *z*-scores were calculated using the mean and standard deviation for each gene from the training set. The R software that implements the method described above is available (http://www.broad.mit.edu/cancer/pub/Revlimid) and as a GenePattern module.

Statistical significance of the predictor was assessed using the proportional chance criterium [19]. Details of this calculation are included in the Text S1.

### Culture of Primary Hematopoietic Progenitor Cells

Cryopreserved human adult bone marrow CD34^+^ cells from normal, healthy donors were obtained from Cambrex (Poietics; Cambrex). Cells were cultured in Serum Free Expansion Medium (SFEM; Stem Cell Technologies) supplemented with 100 U/ml penicillin/streptomycin, 2 mM glutamine, and 40 μg/ml lipids (Sigma). Erythroid differentiation was induced in vitro in two steps [20]. For the first 7 d, cells were cultured in the presence of 100 ng/ml stem cell factor (SCF), 10 ng/ml interleukin-3 (IL-3), 1 μM dexamethasone, and 0.5 IU/ml erythropoietin (Epo). After 7 d, dexamethasone was withdrawn and cells were cultured in the same medium supplemented with 3 IU/ml Epo.

To support both erythroid and megakaryocytic differentiation within the same liquid culture, cells were cultured in the presence of 50 ng/ml thrombopoietin, 100 ng/ml SCF, 10 ng/ml Il-3, 10 ng/ml IL-6, and 0.5 U/ml Epo. The concentration of Epo was increased to 3 IU/ml on day 7. To support both erythroid and myeloid differentiation within the same liquid culture, cells were cultured in the presence of 25 ng/ml SCF, 10 ng/ml IL-3, 40 ng/ml flt-3 ligand, 15 ng/ml granulocyte colony stimulating factor (G-CSF), and 0.5 IU/ml Epo. The concentration of Epo was increased to 3 IU/ml on day 7. Cells were grown in the presence or absence of lenalidomide (Celgene Corporation).

### Flow Cytometry

Cells were harvested for flow cytometry following 10 d of liquid culture. Approximately 5 × 10^5^ cells were labeled with phycoerythrin or FITC-conjugated antibodies for 15 min on ice. Hematopoietic differentiation was assessed using antibodies specific for the erythroid marker glycophorin A (Gly-A, CD235a, Clone GA-R2; BD-Pharmingen), the megakaryocyte marker CD41a (Clone HIP8; BD-Pharmingen), and the myeloid marker CD11b (Clone D12; BD-Pharmingen).

### Methylcellulose Colony Assays

Following 72 h of liquid culture in the presence or absence of lenalidomide, cells were plated on methylcellulose containing Epo, SCF, GM-CSF, and IL-3 (H4434; Stem Cell Technologies). Colony formation was evaluated following 14 d of culture at 37 °C in a humidified atmosphere with 5% CO_2_. Colonies containing greater than 100 erythroid cells were scored as burst-forming unit erythroid colonies (BFU-E), and colonies with greater than 20 granulocytes and monocytes were scored as granulocyte-monocyte colony-forming units (CFU-GM).

## Results

### Identification of a Lenalidomide Response Signature

Lenalidomide is an effective treatment for MDS patients with 5q deletions, but the response rate among patients lacking the deletion is much lower (26%). Moreover, the likelihood of response cannot be accurately determined on the basis of clinical parameters or known molecular assays. In order to develop a predictor of lenalidomide response, we examined the genome-wide gene expression profiles of bone marrow aspirate mononuclear cells obtained from patients with MDS prior to therapy with lenalidomide (Table 1). Responders did not vary significantly from nonresponders with respect to clinical characteristics such as age, bone marrow cell counts, or peripheral blood cell counts (Table 2 and the regression model in Text S1).

**Table 1:**
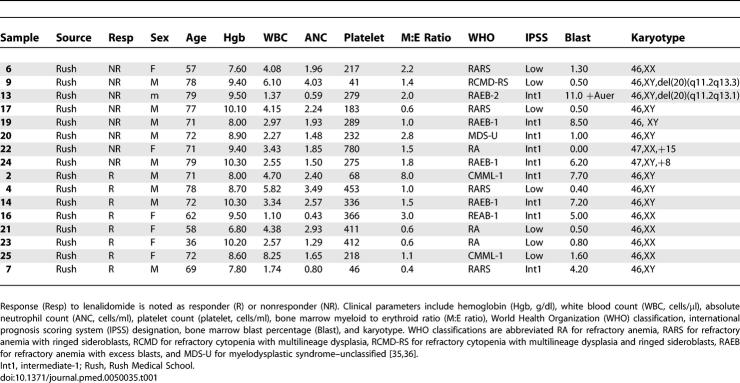
Clinical Parameters for the Samples That Comprise the Training Set

**Table 2:**
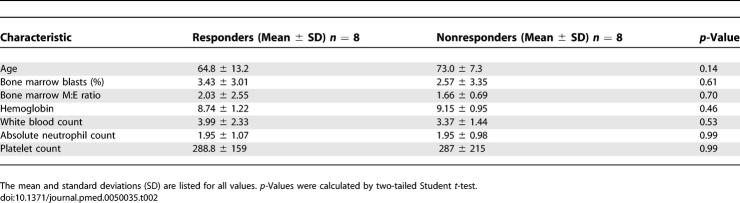
Comparison of the Clinical Characteristics of Lenalidomide Responders versus Nonresponders in the Training Set

**Figure 1:**
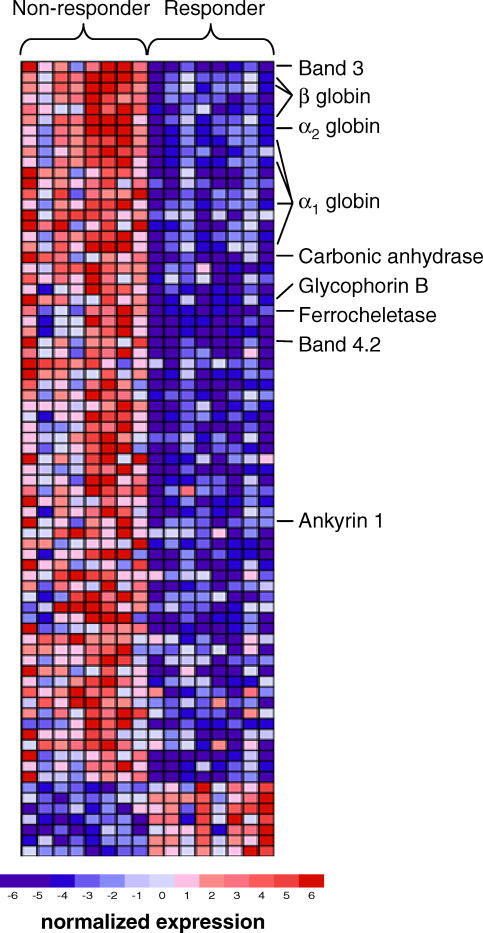
Gene Expression Signature of Lenalidomide Responsiveness Rows correspond to the expression of different genes, and columns correspond to different patient samples. The highest expression of each gene across the samples is portrayed in dark red while the lowest expression is shown in deep blue. Genes with well-documented roles in erythroid differentiation are noted. All genes are listed in Table S2 along with their associated signal-to-noise values, FDR, and fold change between responders and nonresponders.

We compared the gene expression profiles of responders and nonresponders to identify genes that correlate with lenalidomide response in patients lacking 5q deletions. Statistical significance was estimated by permuting the class labels, and the FDR was calculated to correct for multiple hypothesis testing. As shown in Figure 1, 68 probe sets representing 47 genes were more highly expressed in nonresponders than responders (at FDR ≤ 0.10). A total of seven probe sets representing six genes were more highly expressed in responders (FDR ≤ 0.10).

To confirm the differential expression of a subset of the genes using a different platform, we examined the mRNA levels of seven signature genes in the same samples using ligation-mediated amplification followed by detection on fluorescent beads. This assay provides a quantitative detection of each gene in a single multiplexed assay (Figure S1) [21]. As in the microarray data, each signature gene was expressed at higher levels in nonresponders than in responders (Figure S2). These results suggest that a signature of lenalidomide response is detectable.

### Biological Characterization of the Response Signature

To gain insight into the biological meaning of the lenalidomide response signature, we used GSEA to examine the expression of a compendium of 1,688 annotated gene sets representing diverse functional pathways, and asked whether any of these gene sets were enriched in responder versus nonresponder samples. The gene sets most significantly enriched in the nonresponder class included those related to erythroid differentiation. Specifically, a set of genes targeted by the canonical erythroid-specific transcription factor GATA-1 showed strong enrichment, as did a gene set representing targets of STAT5A, a transcription factor activated by the erythroid-specific erythropoietin receptor. Gene sets with the most significant enrichment in both responder and nonresponder samples are listed in Table S4. Consistent with the GSEA result, many of the signature genes are known to be erythroid specific, including genes encoding globin proteins, red blood cell membrane proteins, and enzymes critical for heme biosynthesis (Figure 1). Based on these observations, we hypothesized that the lenalidomide response signature was primarily one of erythroid differentiation.

**Figure 2:**
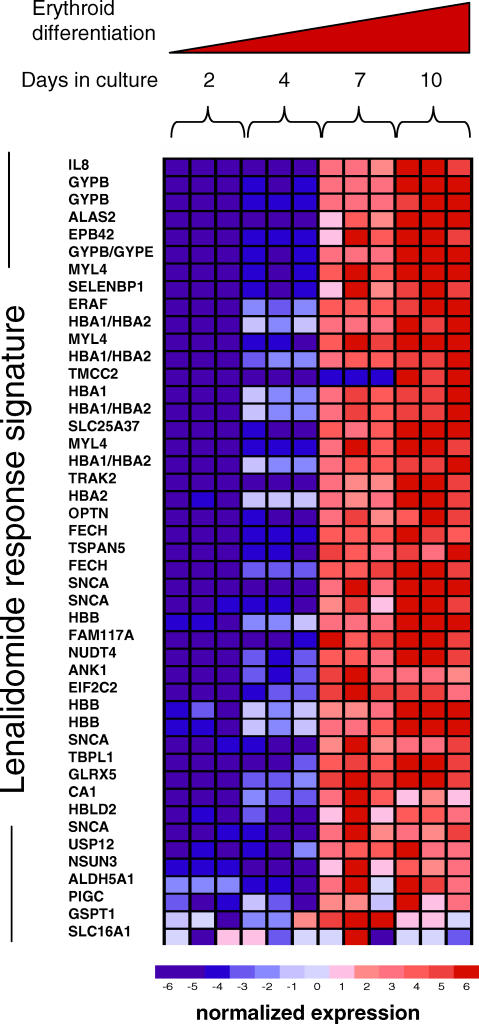
Expression of the Lenalidomide Response Signature during Erythroid Differentiation Gene expression profiles were obtained from human adult bone marrow CD34^+^ cells undergoing erythroid differentiation in vitro at days 2, 4, 7, and 10. Genes are ranked in order of differential expression between day 2 versus day 10. The genes in this signature are coordinately expressed during erythroid differentiation, from lower expression (blue) in immature progenitor cells to higher expression (red) in terminal erythroid differentiation. Some of the genes from Figure 2 are not present in Figure 3 due to the greater number of probe sets on HG_U133 Plus 2.0 microarrays than HG_U133AAofAv2 microarrays. All genes are listed in Table S3.

To validate the erythroid nature of the lenalidomide response signature, we hypothesized that the response signature should be induced as hematopoietic progenitor cells are induced to differentiate along the erythroid lineage in vitro. Accordingly, we monitored the expression of the response signature in primary human CD34^+^ hematopoietic progenitor cells from normal adult bone marrow undergoing erythroid differentiation using a two-phase liquid culture system. Strikingly, the expression of all but one of the signature genes increased dramatically during terminal erythroid differentiation (Figure 2). This result further indicates that the lenalidomide response signature relates to erythroid differentiation.

**Figure 3:**
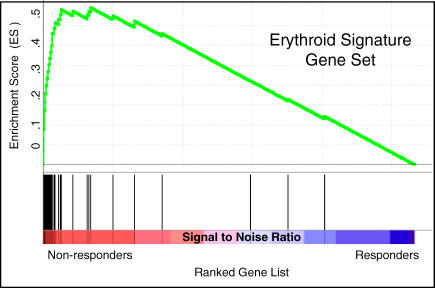
Gene Set Enrichment Analysis Using the Response Signature Expression of the response signature, defined in the training set of samples, was analyzed in an independent test set of samples from MDS patients without 5q deletions. GSEA is a nonparametric statistical methodology that examines whether a set of genes, e.g., the response signature, occur towards the top of a list of genes that is ranked according to a class distinction. In this case, all genes on the microarray were ranked by signal-to-noise ratio in order of their differential expression between nonresponders and responders. Black bars at the bottom of the figure indicate the location of genes in the response signature within the ranked list. The running enrichment score is shown in green. Nearly all of the genes in the erythroid response are expressed more highly in the non-responder class and are at the top of the ranked list of genes, resulting in a strong and statistically significant enrichment score (*p* < 0.001).

### Evaluation of the Lenalidomide Response Signature in an Independent Set of Samples

One important challenge in the development of gene expression-based predictors is ensuring that the predictors continue to perform well when applied to an independent set of samples. We therefore examined the behavior of the erythroid differentiation signature (Table 3) in a new collection of MDS patients for whom lenalidomide response was known. The test set included samples from an additional institution and included samples with and without 5q deletions (Table 4). Among the patients without 5q deletions, the test set had a higher proportion of patients with low-risk International Prognostic Scoring System (IPSS) scores than the training set (62% versus 44%).

**Table 3:**
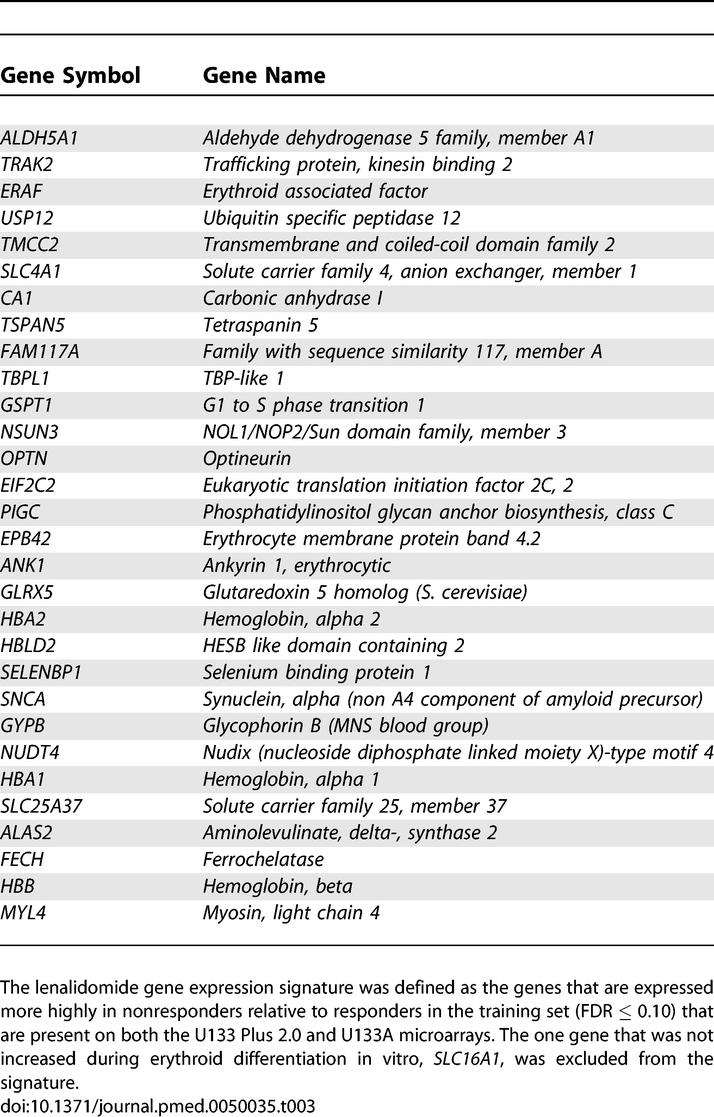
Lenalidomide Gene Expression Response Signature

**Table 4:**
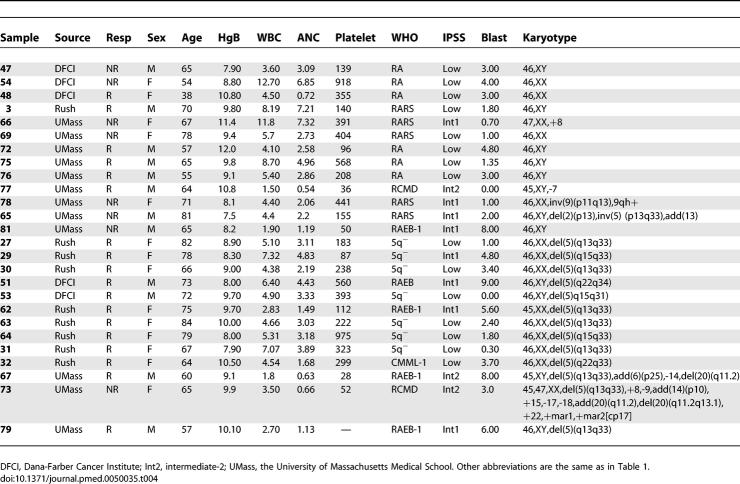
Clinical Parameters for the Samples That Comprise the Test Set

GSEA indicated dramatic enrichment in responding versus nonresponding patients across 26 patients (*p* < 0.001). Importantly, the signature was highly correlated with response among 13 patients lacking 5q deletions (*p* < 0.001) (Figure 3). These findings are consistent with our initial observation that decreased expression of the erythroid differentiation signature correlates with lenalidomide response.

### Development of a Lenalidomide Response Predictor

**Figure 4:**
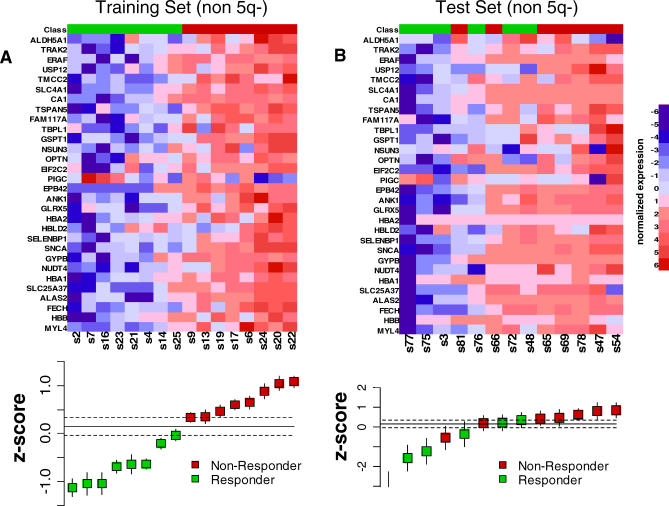
Prediction of Lenalidomide Response Using the Response Signature Each gene in the response signature was normalized to a reference signature and converted to a *z*-score. The heat maps depict the normalized expression of each gene in the response signature. High expression is shown in red, and low expression is shown in blue. The box-and-whisker plots below the heat maps depict the average *z*-scores for the response signature for each sample. The dotted lines indicate the zone in which no call can be made between responder and nonresponder. In the training set (A), all samples are correctly separated by the predictor. In the test set (B), lenalidomide response is correctly predicted nine out of 11 samples (82%), and no call could be made in two samples.

We next sought to create a single score that could be used to evaluate the expression of the response signature in individual patients (as opposed to the dataset as a whole). To create a robust score that is independent of the microarray platforms, we normalized each gene to a panel of five control genes, calculated a *z*-score for each gene, and computed an average *z*-score for all genes in the sample (see Materials and Methods). This average *z*-score metric accurately separated responders from nonresponders in the training set (Figure 4A). The training set was also used to define a “no call” zone representing predictions of low confidence (Figure 4A). This metric also separated responders from nonresponders using the gene expression values obtained from a multiplexed PCR-based assay (Figure S3).

**Figure 5:**
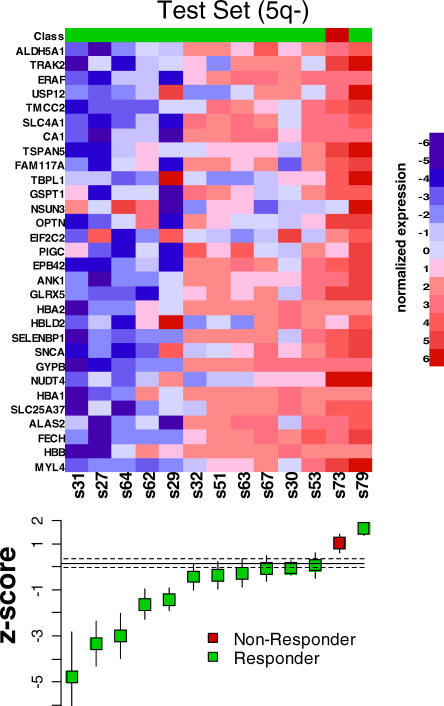
Prediction of Lenalidomide Response in a Cohort of MDS with 5q Deletions Normalized expression of each gene in the response signature is shown in the heat map. The box-and-whisker plots depict the average *z*-score for each sample. Response to lenalidomide was correctly predicted in ten out of 11 samples (91%).

We next applied the predictor without further modification to the 26 validation samples. High-confidence predictions were made on 22/26 patients (85%), 19 of which correctly predicted lenalidomide response (86%)—far beyond what would be expected by chance (*p* = 0.0015). The predictor was similarly accurate within the subset of patients lacking 5q deletions (Figure 4B): high-confidence calls were made in 11/13 patients, and 9/11 (82%) were classified correctly (*p* = 0.0186). Similarly, the predictor was highly accurate among patients harboring 5q deletions (10/11 predicted correctly, Figure 5). Importantly, the one 5q^−^ patient that failed to respond to lenalidomide was correctly predicted to be a nonresponder, suggesting that a common molecular phenotype (an erythroid differentiation defect) underlies all responses to lenalidomide.

The demonstration that the gene expression signature correctly predicted therapeutic response in an independent set of samples acquired from multiple institutions validates the finding that decreased expression of a set of genes that are specific to terminal erythroid differentiation is associated with response to lenalidomide.

### Hematopoietic Effects of Lenalidomide In Vitro

**Figure 6:**
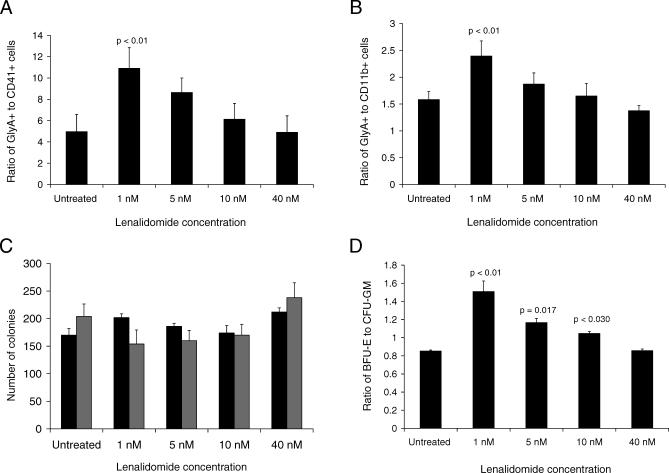
Effects of Lenalidomide on Hematopoietic Differentiation In Vitro Primary human bone marrow CD34^+^ cells were treated with lenalidomide. (A) In cells cultured with cytokines that support erythroid and megakaryocytic differentiation, low doses of lenalidomide increased the number of erythroid (GlyA^+^) cells relative to megakaryocytic (CD41^+^) cells as assessed by flow cytometry. (B) In cells cultured with cytokines that support erythroid and myeloid differentiation, low doses of lenalidomide increased the number of erythroid (GlyA^+^) cells relative to megakaryocytic (CD11b^+^) cells as assessed by flow cytometry. (C) Cells treated with lenalidomide were cultured for 3 d in liquid culture and then plated on methylcellulose. The total number of colony-forming units did not change significantly. As shown in (D), the ratio of erythroid (BFU-E) to myeloid (CFU-GM) colonies increased with low doses of lenalidomide. All *p*-values were calculated by two-tailed Student *t*-test. Confidence intervals for all measurements are portrayed in Figure S4.

The mechanism of action of lenalidomide in MDS is not known, but immunomodulatory and antiangiogenic effects have been observed in other systems [22]. Our observation that an erythroid differentiation signature predicts response, however, suggested an alternative hypothesis, namely that lenalidomide has direct effects on the erythroid potential of hematopoietic cells. To test this hypothesis, we treated primary human adult bone marrow cells with lenalidomide in vitro and evaluated the effects on hematopoietic differentiation. In liquid culture containing cytokines that support erythropoiesis, megakaryopoiesis, and myelopoiesis, low doses of lenalidomide consistently promoted erythropoiesis in culture (Figure 6A and 6B).

To determine whether lenalidomide activates the response signature, we performed gene expression profiling on normal CD34^+^ cells cultured in the presence or absence of lenalidomide for 24 h. The response signature had significantly higher expression in lenalidomide-treated cells (*p* < 0.001 by GSEA, Figure S3).

In addition, we cultured adult human bone marrow cells on methylcellulose following 3 d of treatment with lenalidomide in liquid culture. Lenalidomide significantly increased the ratio of erythroid to granulocyte-monocyte colony-forming units (Figure 6C and 6D). Thus, both liquid culture and methycellulose colony assays demonstrate that lenalidomide promotes erythropoiesis, consistent with the finding that the patients who respond to the drug have the greatest defect in erythroid differentiation.

## Discussion

MDS is characterized by dysplastic differentiation and apoptosis of hematopoietic progenitor cells for one or more lineages [23–25]. Lenalidomide is known to induce hematologic and cytogenetic responses in MDS patients with deletions of Chromosome 5q, leading to its recent approval by the Food and Drug Administration for these particular patients [3–5]. Unfortunately, most MDS patients lack the 5q^−^ abnormality, and lenalidomide response in these patients is estimated at only 26%. Given the considerable side effects of lenalidomide, identifying predictors of drug response is of critical importance. We found that mononuclear cells from bone marrow aspirates of patients who respond to lenalidomide have decreased expression of genes that are specific to terminal erythroid differentiation, regardless of the presence or absence of a 5q deletion. Moreover, we found that lenalidomide acts directly on hematopoietic progenitor cells to increase erythropoiesis relative to other lineages.

The erythroid specificity of the lenalidomide response signature is consistent with the biology of the 5q^−^ syndrome in MDS. Patients with the 5q^−^ syndrome have a severe anemia, a normal or elevated platelet count, and a relatively preserved neutrophil count [26,27]. The gene or genes on Chromosome 5q that block erythroid differentiation have not been identified [26,28–30]. Our gene expression data indicate that lenalidomide–responsive MDS patients lacking 5q deletions have a defect in erythroid differentiation analogous to the ineffective erythropoiesis in patients with 5q deletions, indicating a possible commonality in the molecular basis of MDS in patients who respond to lenalidomide.

The bone marrow of patients with MDS is heterogeneous, containing cellular populations with different genotypes and differentiation states. In some studies, this heterogeneity has been partially mitigated by studying CD34^+^/CD38− cells or the entire CD34^+^ population. Variability in the purification of these populations would likely complicate a gene expression-based clinical diagnostic assay. Our analysis of bone marrow mononuclear cells provides a quantitative integration of the gene expression for cells in all states of differentiation. Specifically, we found decreased levels of genes expressed during terminal erythroid differentiation, a finding that would likely be less evident in CD34^+^ cells.

Lenalidomide was developed as an analog of thalidomide [22], reported to have immunomodulatory, anticytokine, and antiangiogenic properties [31–34], but the mechanism of lenalidomide activity in MDS has not been determined. Clinically, lenalidomide increases red blood cell production and causes neutropenia and thrombocytopenia [3,4]. Our studies show that lenalidomide increases erythropoiesis relative to myelopoiesis and megakaryopoiesis in vitro in the absence of the bone marrow stromal cells that contribute to the bone marrow microenvironment, suggesting that the lenalidomide response is cell autonomous. Therapeutic responses might be due to the promotion of differentiation or the induction of apoptosis in cells bearing mutations that disrupt normal erythropoiesis.

We have found that lenalidomide promotes erythropoiesis and is most efficacious in MDS patients with a block in erythroid differentiation. Whereas most MDS patients with 5q deletions are likely to be treated with lenalidomide, our results suggest that those patients without 5q deletions who are likely to respond can be identified prior to treatment using the erythroid gene expression signature. Notably, despite the heterogeneity of MDS marrows, the signature was predictive in total marrow mononuclear cells, further suggesting that the approach could be feasibly implemented in the clinical setting. These results suggest that further testing of the predictive signature is warranted. These studies also suggest that lenalidomide therapy be considered for other disorders characterized by defects in erythroid maturation, such as Diamond-Blackfan Anemia.

## Supporting Information

Figure S1 Evaluation of the Lenalidomide Response Signature by Ligation-Mediated Amplification with Detection on Fluorescent Microspheres (A) As shown in the schematic diagram, first strand cDNA is generated from oligo-dT primers. Ligation-mediated amplification (LMA) probes that bind to adjacent sequences on a target cDNA are ligated. Annealed probes from all genes are amplified in a single multiplexed PCR reaction using T3 and cT7 primers. The cT7 primer is biotinylated, and amplicons are labeled with streptavidin-coated phycoerythrin (SAPE). The upstream primer for each gene has a unique FlexMAP tag that is recognized by a capture probe on a microsphere of unique fluorescent color.
(103 KB PPT)

Figure S2 The Expression of Seven Genes in the Training Set of Samples Was Determined by LMA Followed by Quantitation of Amplicon Abundance on Luminex Microspheres Consistent with the gene expression profiles determined by Affymetrix microarrays, the erythroid signature is expressed at lower levels in all nonresponders than in responders. As with the microarray data, the response of all samples was correctly predicted by gene expression.
(109 KB PPT)

Figure S3 Gene Set Enrichment Analysis Using the Lenalidomide Response Signature in Lenalidomide-Treated Cells CD34^+^ cells were cultured in the presence 1 nM lenalidomide or an untreated control for 24 h. Each condition was performed in triplicate. The expression of the lenalidomide response signature was examined in these samples using GSEA. All genes on the microarray were ranked by signal-to-noise ratio in order of their differential expression between lenalidomide treatment and the untreated control. Black bars at the bottom of the figure indicate the location of genes in the response signature within the ranked list. The running enrichment score is shown in green. Nearly all of the genes in the lenalidomide response signature are expressed more highly in the lenalidomide-treated cells and are at the top of the ranked list of genes, resulting in a strong and statistically significant enrichment score (*p* < 0.001).
(89 KB PPT)

Figure S4 Confidence Intervals for the Effects of Lenalidomide on Hematopoietic Differentiation In Vitro The results from Figure 6 in the main text are portrayed as a box-and-whisker plot. The horizontal bars represent the median values, rectangles represent the interquartile ranges, and error bars represent the 95% confidence intervals. Primary human bone marrow CD34^+^ cells were treated with lenalidomide.
(A) In cells cultured with cytokines that support erythroid and megakaryocytic differentiation, low doses of lenalidomide increased the number of erythroid (GlyA^+^) cells relative to megakaryocytic (CD41^+^) cells.
(B) In cells cultured with cytokines that support erythroid and myeloid differentiation, low doses of lenalidomide increased the number of erythroid (GlyA+) cells relative to megakaryocytic (CD11b^+^) cells.
(C) Cells treated with lenalidomide were cultured for 3 d in liquid culture and then plated on methylcellulose. The total number of colony-forming units did not change significantly. As shown in (D), the ratio of erythroid (BFU-E) to myeloid (CFU-GM) colonies increased with low doses of lenalidomide.
(87 KB PPT)

Text S1 An Erythroid Differentiation Signature Predicts Response to Lenalidomide in Myelodysplastic Syndrome (54 KB DOC)

Table S1 List of All .cel Files for Microarray Experiments (30 KB XLS)

Table S2 Significant Markers of Responders and Nonresponders from Figure (56 KB XLS)

Table S3 Lenalidomide Response Signature (28 KB XLS)

Table S4 Gene Sets Enriched in Responders and Nonresponders in Test Set (Figure 3) (26 KB XLS)

## Abbreviations

BFU-E: burst forming unit–erythroid
CFU-GM: colony forming unit–granulocyte/monocyte
CMML: chronic myelomonocytic leukemia
Epo: erythropoietin
FDR: false-discovery rate
GSEA: gene set enrichment analysis
IL: interleukin
IPSS: International Prognostic Scoring System
MDS: myelodysplastic syndrome
SCF: stem cell factor
