# Supplementary material for: An Erythroid Differentiation Signature Predicts Response to Lenalidomide in Myelodysplastic Syndrome
Source: PLoS Med. 2008 Feb 12;5(2):e35. doi: 10.1371/journal.pmed.0050035 (PMC2235894; doi:10.1371/journal.pmed.0050035)
Supplement: Supplementary file 1 [file pmed.0050035.sd001.doc]

**Supplementary information Text S1**

**An erythroid differentiation signature predicts response to lenalidomide in Myelodysplastic Syndrome**

**Benjamin L. Ebert, Naomi Galili, Pablo Tamayo, Jocelyn Bosco, Raymond Mak, Jennifer Pretz, Christine Ladd-Acosta, Richard Stone, Todd R. Golub, Azra Raza**

**Contents:**

1. Regression analysis of clinical characteristics	p. 1

2. p-value calculation for the lenalidomide response predictor	p. 3

**1. Analysis of the predictive value of clinical characteristics in the training set**

In order to assess the predictive power of several clinical characteristics on Lenalidomide response, we built an explicit regression model using them as inputs. The clinical characteristics considered were gender, age, hemoglobin (HgB), white blood count (WBC), absolute neutrophil count (ANC), platelet count (platelet), myeloid to erythroid ratio (M.E.ratio), International Prognostic Score (IPSS) and bone marrow blast count (blast). We fit a logistic regression model using those 9 characteristics as regressor variables using the “glm” R function.^1^ We analyzed the results using sequential analysis of deviance using the “anova.glm” R function. The following table summarizes the results:

|  | **Sequential Analysis of Deviance** | | |
| --- | --- | --- | --- |
| **Term** | **Deviance Residual** | **Df** | **P(>\|Chi\|)** |
| Intercept |  | 15 |  |
| Gender | 1.1 | 14 | 0.3 |
| Age | 1.7 | 13 | 0.19 |
| HgB | 0.17 | 12 | 0.68 |
| WBC | 1.4 | 11 | 0.23 |
| ANC | 0.79 | 10 | 0.37 |
| Platelet | 1.2 | 9 | 0.28 |
| M.E.ratio | 0.14 | 8 | 0.7 |
| IPSS | 0.27 | 7 | 0.61 |
| Blast | 0.026 | 6 | 0.87 |

The columns correspond to the deviance residuals, degrees of freedom and sequential χ^2^ test score. As seen in the χ^2^ scores, none of these clinical characteristics are statistically significant individually, or collectively, as Lenalidomide response predictors. However, if we include the response signature for the multiplexed PCR data (z.vector.signature) as a regressor variable in the model, it is highly significant. The following table summarizes those results:

|  | **Sequential Analysis of Deviance** | |  |
| --- | --- | --- | --- |
| **Term** | **Deviance Residual** | **Df** | **P(>\|Chi\|)** |
| Intercept | 22 |  |  |
| Gender | 1.1 | 14 | 0.3 |
| Age | 1.7 | 13 | 0.19 |
| HgB | 0.17 | 12 | 0.68 |
| WBC | 1.4 | 11 | 0.23 |
| ANC | 0.79 | 10 | 0.37 |
| Platelet | 1.2 | 9 | 0.28 |
| M.E.ratio | 0.14 | 8 | 0.7 |
| IPSS | 0.27 | 7 | 0.61 |
| Blast | 0.026 | 6 | 0.87 |
| z.vector.signature | 15 | 5 | 0.0001 |

**2. Details of the p-value calculation for the *lenalidomide response predictor* using the proportional chance criterion.**

We followed the standard methodology to compute the significvance of a predictor by comparing it with the proportional chance hit rate of a random predictor (diagonal) in the confusion matrices of predictor calls (excluding no-calls). For more background see for example chapter 16 of Huberty and Olejnik [19]

_________________________________________________________________________

Prediction of non 5q- test samples by the *lenalidomide response predictor:*

Predicted:

NR	R	Total Call	No-Call	Total

Actual: NR	5	1	6	1	7

R	1	4	5	1	6

Total	6	5	11	2	13

Cpro = (6/11) * (6/11) + (5/11) * (5/11) = 0.5041 (hit rate on diagonal by chance)

Cmodel = 5/11 + 4/11 = 9/11 = 0.8182 (hit rate on diagonal attained by model)

z = (Cmodel - Cpro)/sqrt(Cpro*(1-Cpro)/11) = 2.083

p-val = 0.0186 (assuming a normal distribution for z)

Confidence interval for number of correct calls (5%, 95%) = (2.8, 8.3) model attains 9

p-val = 0.00632 (assuming a binomial distribution with N=11, q=Cmodel and prob=Cpro)

________________________________________________________________________

Prediction of all test samples (non 5q- and 5q-) by the *lenalidomide response predictor:*

Predicted:

NR	R	Total Call	No-Call	Total

Actual: NR	6	1	7	1	8

R	2	13	15	3	18

Total	8	14	22	4	26

Cpro = (8/22) * (7/22) + (14/22) * (15/22) = 0.5496 (hit rate on diagonal by chance)

Cmodel = 6/22 + 13/22 = 19/22 = 0.8636 (hit rate on diagonal attained by model)

z = (Cmodel - Cpro)/sqrt(Cpro*(1-Cpro)/22) = 2.961

p.val = 0.0015 (assuming a normal distribution for z)

Confidence interval for number of correct calls (5%, 95%) = (8.3, 15.9) model attains 19

p-val = 0.00033 (assuming a binomial distribution with N=22, q=Cmodel and prob=Cpro)
